# Unexpected High Genetic Diversity at the Extreme Northern Geographic Limit of *Taurulus bubalis* (Euphrasen, 1786)

**DOI:** 10.1371/journal.pone.0044404

**Published:** 2012-08-30

**Authors:** Vítor C. Almada, Frederico Almada, Sara M. Francisco, Rita Castilho, Joana I. Robalo

**Affiliations:** 1 Eco-Ethology Research Unit, ISPA University Institute, Lisboa, Portugal; 2 Centro de Ciências do Mar (CCMAR, CIMAR- Associate Laboratory), Universidade do Algarve, Campus de Gambelas, Faro, Portugal; Biodiversity Insitute of Ontario - University of Guelph, Canada

## Abstract

The longspined bullhead (*Taurulus bubalis,* Euphrasen 1786) belongs to the family Cottidae and is a rocky shore species that inhabits the intertidal zones of the Eastern Atlantic since Iceland, southward to Portugal and also the North Sea and Baltic, northward to the Gulf of Finland, with some occurrences in the northern Mediterranean coasts eastward to the Gulf of Genoa. We analysed the phylogeographic patterns of this species using mitochondrial and nuclear markers in populations throughout most of its distributional range in west Europe. We found that *T. bubalis* has a relatively shallow genealogy with some differentiation between Atlantic and North Sea. Genetic diversity was homogeneous across all populations studied. The possibility of a glacial refugium near the North Sea is discussed. In many, but not all, marine temperate organisms, patterns of diversity are similar across the species range. If this phenomenon proves to be most common in cold adapted species, it may reflect the availability of glacial refugia not far from their present-day northern limits.

## Introduction

One of the major concerns in studies of phylogeography of northeastern Atlantic organisms has been the location of unglaciated refugia, where marine inshore organisms could survive the successive glacial peaks of the Pleistocene (the last glaciation ended about 10–11 thousand years ago, kya). These refugia must have acted as propagule sources for the recolonization of more northerly areas during the interglacials [1]. This effort revealed a variety of potential refugia that, depending on the species studied, range from the Mediterranean, southwest Europe, waters around southwest Ireland and Southwest Britain, the Hurd Deep (a saline lake that existed near the entrance to the North Sea during glacial times) to areas near the Faeroes, north Norway and Iceland (for a review see [2]). Virtually all areas that were unglaciated during glacial phases may have acted as refugia for some species. In the last glaciation, for which paleoclimatic data are available in greater detail, it is known that the North Sea was basically glaciated and had almost no habitats suited for marine organisms [3], [4]. For the northeastern Atlantic some authors [5], [6] proposed that the polar front could have shifted to the South, reaching western Portugal. More conservative authors suggested that permanent ice sheets could be restricted to more northern limits, namely north of the Bay of Biscay [4]. At the Last Glacial Maximum (LGM), the Sea Surface Temperature (SST) off west Portugal in the summer was similar to the modern one, but during cold pulses, specially to the end of the glaciation, temperatures as low as 5°C occurred in the area [7]. Despite of this huge advance of the polar front to the south, many areas north of Biscay are considered to have been unglaciated (some deep trenches in the Western English Channel, Southwest Ireland, Iceland and the Faroe Islands and Northern Norway; [2]).

The inshore fish that now occur in west Europe vary in their thermal tolerance and biogeographic origin. Several distributional patterns can be observed. Warm temperate and tropical eurithermic species rarely breed in the North Sea and must have survived the glaciations in the warmer waters of the Mediterranean, south Iberia and/or western Africa (e.g. most sparids, some blennids and wrasses). More cold tolerant species (cold temperate) presently live and breed north of the English Channel in the North Sea, reaching northern Scandinavia. Artic species occur mainly in or near the Arctic and are very rarely found south of the English Channel [8], [9], [10]. Such a variety of thermal tolerances implies that what constituted a refugium for a species could be too cold to others. These findings raise a number of interesting issues. Cold temperate species could have found much wider areas of suitable habitat well to the south of their present day distributions. Thus, the areas available as refugia may have generated very different patterns of genetic diversity during glacial periods and very different responses during the recolonization process, when milder climates became established. Indeed, a glacial period could represent a contraction for some warm water species, while it could represent an expansion to cold water ones (in parallel to the expansion assumed to have occurred in terrestrial boreal and arctic species, [10]).

When we turn to genetic diversity, the available data reveal, again, many contrasting patterns. Some results conform to a pattern of high level of genetic diversity in the southern (refugial) part of the range, with genetic diversity decreasing to the northern limit of the species distribution. In these cases, few haplotypes will be present; near the northern limit they would be widespread in large areas and often derived from alleles present in the south, private haplotypes being rare or absent. Examples in inshore fish include *Symphodus melops* [11], *Atherina presbyter* [12] *Pomatoschistus microps* [13] and *Syngnathus typhle* [14]. Many other studies, both in fish and invertebrates, do not conform to this pattern, presenting similar depths of genealogies throughout their entire ranges, with no drop of diversity in the extreme north (e.g. *Homarus gammarus* [15]; *Nephrops norvegicus* [16]; *Sprattus sprattus* [17]; *Lipophrys pholis* [18]).

Taking all studies together, the phylogeographic patterns uncovered in the northeastern Atlantic hardly fit a single explanation. We suggest that the differences in thermal tolerances mentioned above may have an important role in explaining a substantial part of the variability in the results. Multiple refugia, varying in latitude and probable SSTs during glacial peaks likely mean that warm water species must have survived in warm areas, while cold temperate species may have survived in low temperature refugia, closer to the ice sheets. In order to assess this hypothesis, however, the number of phylogeographic studies of western European inshore fishes must be increased using fish with contrasting thermal preferences in order to provide data for multi species comparisons.

In the first years of marine phylogeography much emphasis was placed in thermophilic species since much of the focus in studies of European marine ichthyofauna involved the assessment of the role of the Mediterranean as a refugium and the level of differentiation between Atlantic and Mediterranean populations [19]. In recent years increasing numbers of more cold tolerant species are being investigated [17], [20]–[22].

The longspined bullhead (*Taurulus bubalis*, Euphrasen 1786) belongs to the family Cottidae and is a rocky shore species that inhabits the intertidal zones (tide pools and inshore waters) of the Eastern Atlantic from Iceland, southward to Portugal, the North Sea and the Baltic northward to the Gulf of Finland, with some occurrences in the northern Mediterranean coasts eastward to the Gulf of Genoa [9]. Although we could not find a comprehensive phylogenetic study of the Cottidae, [23] placed the family in the order Scorpaeniformes. According to the same author, the family Cottidae includes approximately 275 species primarily distributed in boreal and cold-temperate regions. This family is generally considered to be of recent North Pacific origin [24]–[26], possibly having invaded the Artic/Atlantic when the Bering Land Bridge disintegrated about 5,5 to 3,5 million years ago [10].

*Taurulus bubalis* adults are quite sedentary and must perform restricted movements in a given stretch of rocky shore. The eggs are guarded by the male until hatching, after which larvae enter the plankton for some time [27]. This species has the advantage of dispersing only during the larval stage, removing the movements of adults as a source of dispersal. At the same time, it is a very cold tolerant species common in north European waters but uncommon to the south (e.g. at the latitude of Portugal), where nests and eggs are very rarely observed, especially in warmer years (VC Almada, unpublished results).

Currently the species occurs in waters with SSTs that range from around 2–8°C in Scandinavia to 13–18°C in Portugal (February, data collected in 2007, [28]). In August the corresponding figures are around 3–10°C in Scandinavia to 15–19°C in Portugal. If we compare these temperatures (February and August) with those provided by [29] for 18 kya we find that Scandinavian waters were glaciated while in Portugal the temperatures ranged from 6–12°C (both in February and August).

In the present paper we use one mitochondrial and one nuclear marker to assess the phylogeographic patterns and processes of *Taurulus bubalis* with samples ranging from Central Portugal to Norway and Sweden.

## Results

**Figure 1:**
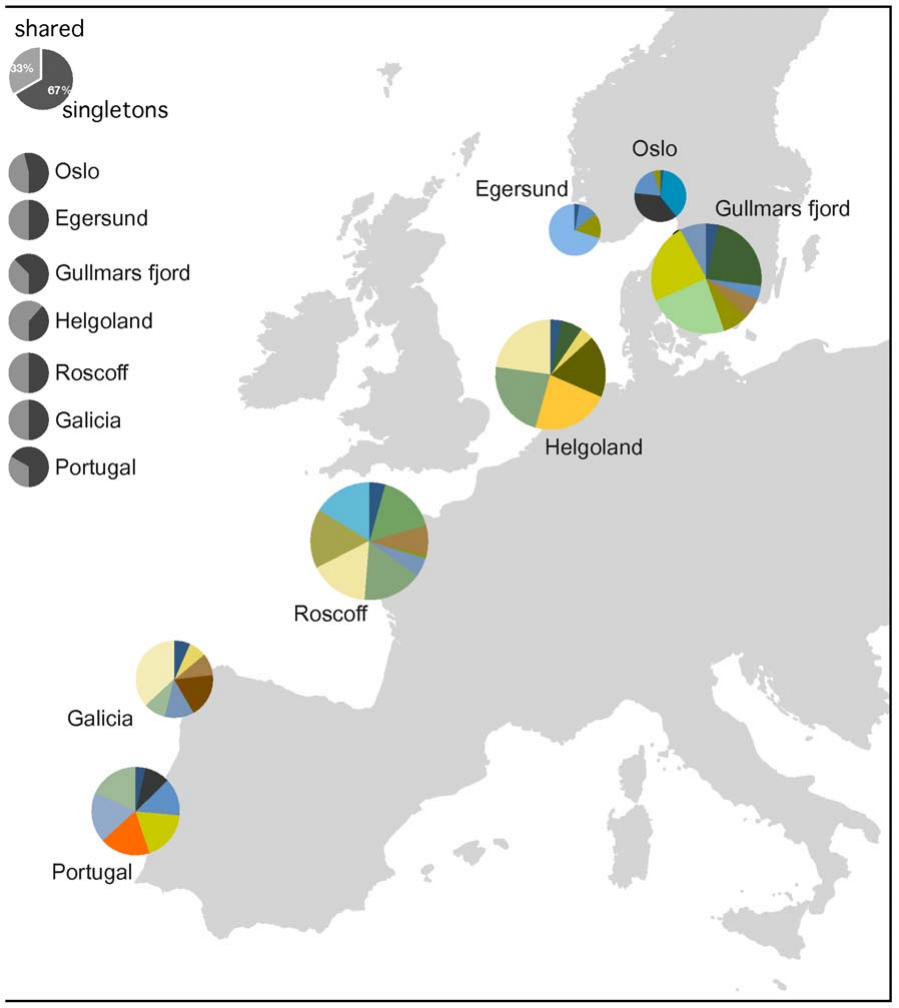
Sampling locations and haplotype distribution for *Taurulus bubalis*. Map with the sampling locations for *Taurulus bubalis*. Top gray circle represents proportion of singleton haplotypes (light gray) and shared between individual haplotypes (dark gray), while smaller gray circles represent the same proportion for each location. Colored circles represent only the relative proportion of shared between individual haplotypes in each location, with each haplotype having a different color. Size of colored circle is proportional to sample size.

The mitochondrial control region (CR) data set consisted of a total of 425 characters (165 individuals, Genbank Accession Numbers JQ013526-686 and JQ061250-53), representing a total of 27 haplotypes (Figure 1). The amplification of the first intron of the S7 gene yielded a fragment of 506 base pairs, with 13 SNPs (149 individuals, Genbank Acession Numbers JQ039746-894). Due to logistical problems we could not amplify the S7 intron for the Egersund samples. Nevertheless, the samples from this location were included in the CR dataset because they show no significant differentiation from the remaining Scandinavian locations.

**Figure 2:**
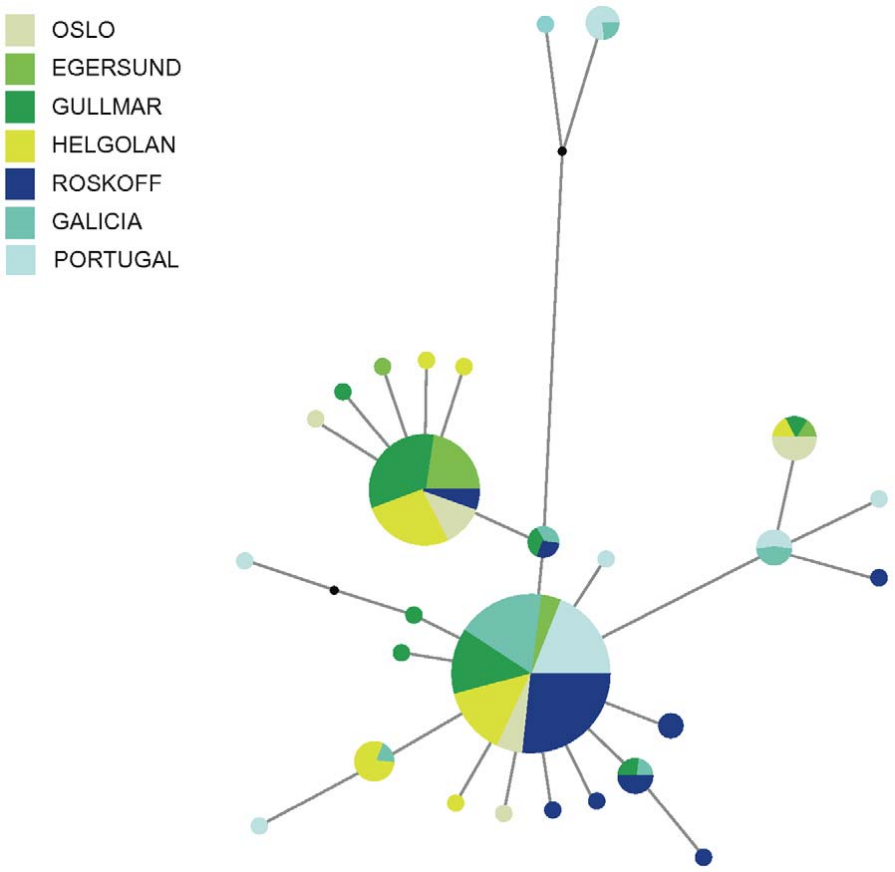
Haplotype network for the mitochondrial control region of *Taurulus bubablis*. Haplotype network for the mitochondrial control region of *Taurulus bubablis.* Colors refer to the region in which haplotypes were found. In the case where haplotypes are shared among regions, shading is proportional to the frequency of the haplotype in each region. The area of the circles is proportional to each haplotype frequency.

The CR haplotype network shows two dominant haplotypes, separated by only two mutational steps (Figure 2). The most common haplotype represents 79 fish from all localities sampled. The haplotypes derived from this one are normally singletons from all localities, only one or a few mutations away. The second most common haplotype (two mutational steps away from the first) represents 40 fish, all from the northern locations (Helgoland, Gullmars fjord, Oslo and Egersund) and Roskoff. This haplotype has fewer derived ones, all from northern localities.

For the CR fragment and S7 intron, the genetic diversity indices for each population are summarized in Table 1. The diversity indices are moderate (when compared with those from other marine fishes of the same geographical areas [11]–[13], [30]) and do not vary along the latitudinal gradient. Inspection of Table 1 shows that the highest diversity values can be found in Atlantic and in Scandinavian populations.

**Table 1:** Sampling locations, haplotypes and diversity measures.

			Mitochondrial DNA control region							first intron of S7 gene			
Locations	Code	Coordinates	N	NH	Haplotype diversity ± s.d.	Nucleotide diversity ± s.d.	Mean number of pairwise difference ± s.d.	Tajima’s D	Fu’s Fs	N	NH	Nucleotide diversity ± s.d.	Mean number of pairwise difference ± s.d.
Oslo	OS	59°17'N/1°38'E	14	5	0.7912±0.0673	0.0066±0.0041	2.7912±1.5684	0.3812	0.9304	14	14	0.0042±0.0027	2.1138±1.2141
Gullmar	GU	58°19'N/11°18'E	30	8	0.6943±0.0586	0.0037±0.0025	1.5655±0.9603	−1.0573	−2.1590	22	19	0.0047±0.0030	2.0687±1.1806
Egersund	EG	58°24'N/6°'E	14	4	0.5714±0.1322	0.0037±0.0026	1.5934±1.0052	−0.5826	0.6309				
Helgoland	HE	54°11′N/7°53'E	30	7	0.7333±0.0580	0.0035±0.0024	1.4828±0.9218	−0.9075	−1.3971	29	11	0.0016±0.0013	0.8282±0.6005
Roscoff	RO	48°43'N/3°59'W	32	9	0.5706±0.1290	0.0027±0.0020	1.1351±0.7567	−**1.7738**	−**4.5322**	37	17	0.0024±0.0017	1.2044±0.7781
Galicia	GA	42°13'N/8°58'W	21	7	0.5619±0.1263	0.0035±0.0024	1.4762±0.9299	−**1.8110**	−2.0638	22	9	0.0024±0.0017	1.1575±0.7620
Portugal	PT	38°28'N/8°58'W	24	7	0.6051±0.1110	0.0058±0.0036	2.4674±1.3815	−0.5499	−0.1996	25	10	0.0033±0.0022	1.6906±1.0068

Sampling locations, coordinates, haplotype numbers and diversity measures for *Taurulus bubalis*’s mitochondrial control region and S7 first intron. Number of individuals per location (N); Number of haplotypes (NH); (in S7, as calculated by Arlequin with the EBL algorithm); Haplotype diversity; Nucleotide diversity; Average number of pairwise differences within populations; Tajima’s *D*; Fu’s *Fs*. Significant values in bold (*P<*0.05).

For the CR data, haplotype diversity values were not significantly different among populations according to the X2 test developed by [31] (*x* ^2^ = 5.930, *p* = 0.431).

SAMOVA (CR dataset) yielded a maximum F_CT_ (0.169; *p* = 0.030) for two groups: the North Sea locations *versus* the three Atlantic locations.

**Figure 3:**
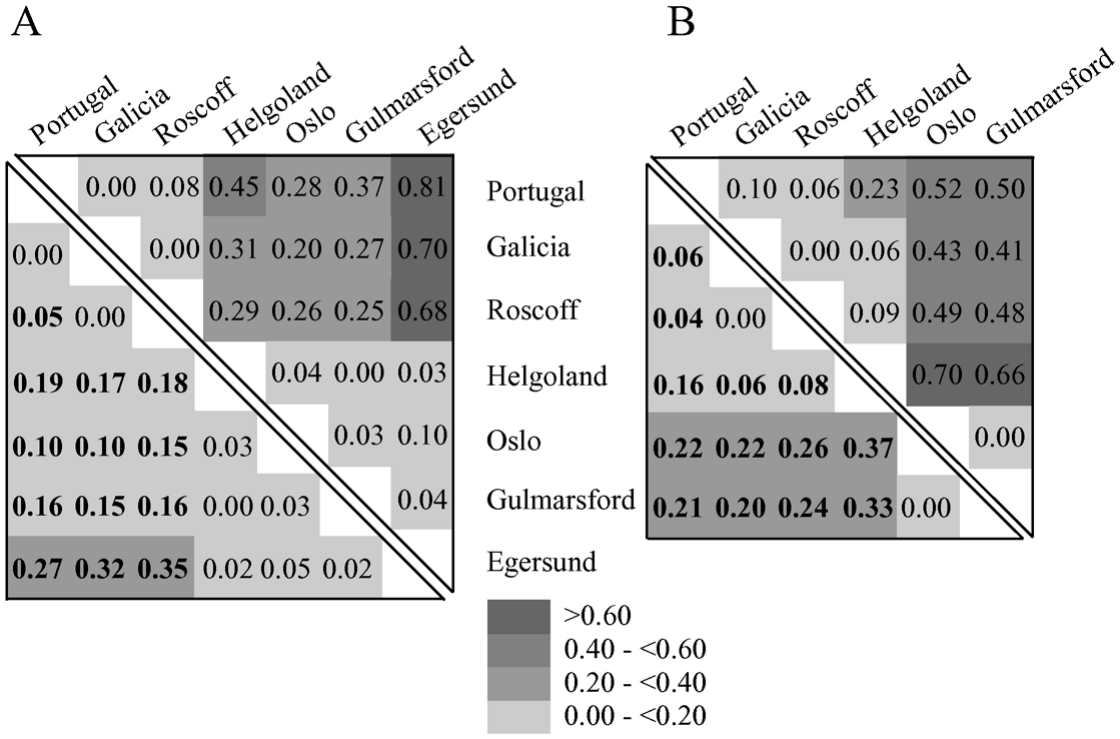
Pairwise Fsts and corrected average pairwise differences for *Taurulus bubalis*. Pairwise *F*_ST_s (below the diagonal) and corrected average pairwise differences (above diagonal) for CR (a) and S7 (b) among *Taurulus bubalis* locations. Significant levels are shaded according to the legend, after correction using the false discovery rate (FDR). Probability values corrected with Qvalue.

The pairwise *F*_ST_s and corrected average pairwise differences shown in Figure 3 (a, CR and b, S7) mostly support the SAMOVA results. In all comparisons FSTs and corrected parwise differences are fully concordant. According to the CR data two groups can be found: one from North Sea (including all samples from Scandinavia and including Helgoland) and a southern one (including all locations South of the North Sea). The North Sea locations are not significantly different among themselves. The same does not apply to southern populations. Although Portuguese and Spanish populations are not significantly different, only the Spanish population (Galicia) is not significantly different from Roscoff, suggesting an isolation by distance scenario. We cannot derive the same conclusions from the S7 gene. The only population pairs that are not significantly different are Galicia-Roscoff and Gullmars fjord – Oslo. These results do not contradict the CR ones but do not support the connection Portugal-Galicia and Helgoland - Scandinavian populations.

Given the concordance between the pairwise *F*_ST_s and SAMOVA we decided to pool together Egersund, Oslo, Gullmar Fjord and Helgoland *vs* Roscoff, Spain and Portugal for further analyses. A hierarchical AMOVA for the S7 data using the same two groups (except for the Egersund sample, not available in S7) yielded a non significant difference among groups with most variation being within populations: percentage of variation among groups = 2.89 (*p* = 0.296); percentage of variation among populations within groups = 14.56 (*p*<0.001) and percentage of variation within populations = 82. 56 (*p*<0.001) (FST = 0.1744).

Tajima and Fu’ Fs tests, for the CR dataset, yielded negative and significant results only for the Roscoff population (Tajima’s D = −1.774, *p*<0.05; Fs = −4.532, *p*<0.05), while for the Galicia population only Tajima test yielded a negative and significant value (Tajima’s D = −1.881, *p*<0.05). In the absence of negative and significant values for the neutrality tests in most populations the application of mismatch analysis was assumed to be inappropriate.

**Figure 4:**
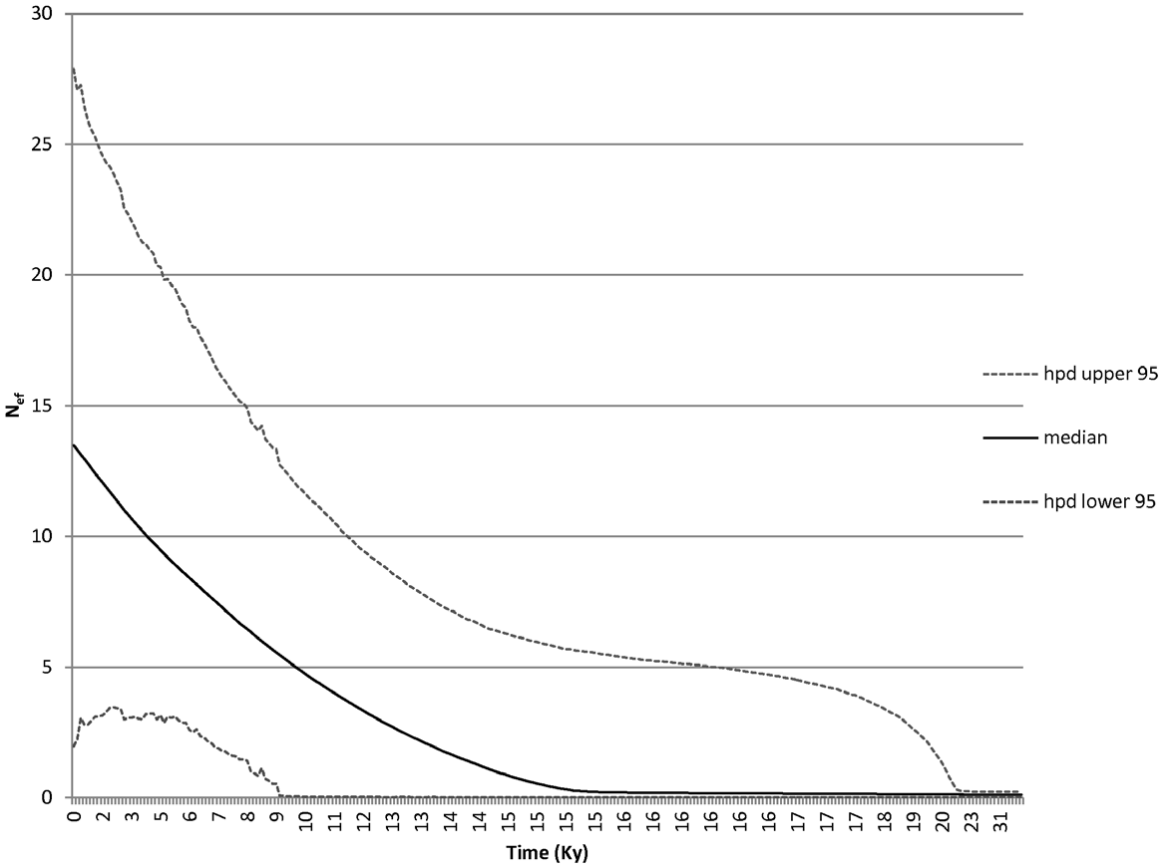
BSP for the CR dataset of *Taurulus bubalis*. Bayesian Skyline Plot for the mitochondrial control region of *Taurulus bubalis*.

Figure 4 shows the BSP for the CR dataset of *T. bubalis.* The plot reveals that the longspined bullhead, as a whole, experienced a faster population growth in the past 15 ky (∼13-fold), reaching a N_ef_ of 13 million fish in the present day. The Time for the Most Recent Common Ancestor_ (_ *t*_MRCA)_ for *T. bubalis* is 12.701 kya, with the southern population having a higher *t*_MRCA_ (6.205 kya) when compared with the northern population (5.425 kya).

Lamarc analyses yielded higher theta for the southern population (average 0.012) in comparison with the northern population (average 0.005), suggesting that the southern population may have a larger N_ef_ than the northern one. The migration rate was higher northwards (average 2,348), than in the opposite direction (average 0,497). We could not perform estimations of population age because the model including growth did not converge.

## Discussion

The results presented above highlight two main features of the phylogeography of *T. bubalis*. The genetic diversity is similar throughout the entire species range and, although there is statistically significant genetic differentiation between the Atlantic and the North Sea, the northern populations display a mixture of widespread haplotypes with others that are almost exclusive of the North Sea.

The presence in the North Sea of a mixture of widely distributed haplotypes with an entire clade almost exclusive of this area, from which a number of private haplotypes derive, is consistent with the hypothesis that the North Sea was colonized from at least two different sources. Indeed, these results are compatible with a scenario with two components: 1) survival in the Atlantic with an expansion to the north as the climate ameliorated after the end of the last glaciation, an expansion that is possibly still in progress; 2) persistence in periglacial conditions of at least one high latitude population that could take advantage of its proximity to the retreating ice to colonize quickly the new habitats that were becoming available. We cannot discard the possibility that the high genetic diversity in the North Sea resulted from an expansion from the Atlantic population with *in situ* accumulation of mutations in the north. This possibility seems however less probable because of the clade which is exclusive to the North Sea and nearby Roscoff represents almost half of the fish sampled in the North Sea (where it is widespread), but gave rise only to 5 haplotypes a single mutational step away.

*T. bubalis* is a cold water adapted species that breeds at very high latitudes (distribution range from 72°N – 35°N, 24°W – 33°E according to [9]) and was hardly found breeding in Portugal after many years of littoral surveys (VC Almada, unpublished). It is likely that all unglaciated parts of west European shores could have served as glacial refugia. In the glacial Mediterranean temperatures would be certainly more favourable to the survival and breeding of this species than they are now, raising the possibility that the Mediterranean populations of *T*. *bubalis* were much larger than today as suggested by [32] for other cold adapted fish species. If this interpretation is correct, many distinct populations of *T*. *bubalis* may have survived, some far from the extreme north and others not far from the North Sea. An expansion and migration from the Atlantic would have enriched the North Sea gene pool. This would reconcile the shallow genealogies, the homogeneity of genetic diversity and the mixed of shared and more local haplotypes present today in both areas. Indeed, the results of BEAST for all samples combined suggest an expansion just after the LGM, continuing in the current interglacial.

Other inshore organisms of West Europe show different patterns of diversity along the shores of the northeastern Atlantic. *S. melops* [11] stands out, like *A. presbyter* [12], *P. microps* [13] and *S. typhle* [14], as examples of high diversity in the southern limit and a sharp decline in genetic diversity in the North of the species range. There is an increasing number of papers reporting cases where diversity remains almost at the same level across the latitudinal range of marine species (e.g. *N. norvegicus* [16]; *H. gammarus* [15]; *P. marinus* [20]; *S. sprattus* [17], *L. pholis* [18]). Initial studies in phylogeography of temperate species [33], [34] postulated that the decline of genetic diversity to the highest latitudes occupied by a species would be the rule. Dispersal, through a series of stepping-stones, would generate a series of bottlenecks that would deplete the variation transported initially from the source population. If, however, in marine organisms (with propagules transported in the plankton) the numbers of founders are very high and travel long distances, a source population may export much of its diversity to the newly colonized areas. In these new areas, if the populations are far from the carrying capacity of the habitat, the bottlenecks may not occur. If, in addition, the dispersal per generation continues to be high, as time passes, the populations may get mixed, erasing any possible signature of the first colonization. A more parsimonious hypothesis to explain high diversity in the northern limit of a species range seems plausible if the phenomenon proves to be especially common in cold adapted species. In cold water species like *T. bubalis* the high genetic diversity in the northern limit of the range may result from the expansion process that adds the polymorphisms of southern populations to some periglacial and glacial persistent populations.

In conclusion, *T. bubalis* exhibits a mixture of shallow genealogy, suggesting an important reduction in the past, some differentiation between North Sea and Atlantic, with likely survival in multiple glacial refugia, combined with appreciable level of haplotype sharing and connectivity and gene flow towards the North Sea. If this hypothesis is correct, the thermophilic species will tend to show reduced diversity in the northern limit of their range, because the northern refugia would not be sufficiently warm to allow survival during glacial peaks, so that the refugia for these fish would occur only in south Europe or even more to the south.

These results must be interpreted with caution for three reasons. First, although we sampled a relevant part of the distribution, some areas remained to be analysed. Special attention must be given in the future to the shores around Britain and Ireland because they provide an alternative connection to the North Sea between northern Scotland and Norway. Second, glaciations involved sea level drops of more than one hundred meters. These lowerings of sea level may have caused changes in the inshore habitats available which are still difficult to model. Third, we used only two markers. Nevertheless, we think that this approach allows a profitable comparison between the pattern found in *Taurulus bubalis* and the ones obtained in several other marine species, due to the broad use of both markers in the literature.

Further studies with organisms with varied life-histories and ecology and population simulations are clearly needed to test the hypothesis raised in this study, on the role of temperature tolerance in shaping the genetic structure observed in European inshore organisms. This is particularly relevant to species like *T. bubalis*, for which the southern part of their range may become unsuitable if sea surface temperatures continue to increase.

## Materials and Methods

### Sampling

A total of 165 samples of *T. bubalis* were collected from 7 locations along West Europe, from Portugal to Norway (Figure 1): Sintra/Cascais (Portugal), Galicia (Spain), Roscoff (France), Helgoland (Germany), Gullmars fjord (Sweden), Oslo and Egersund (both in Norway). Samples from the North Sea and Roskoff came from beach-seeing surveys by Institute of Marine Research (Norway), Alfred Wegener Institute (Germany) and Station Biologique de Roscoff (France - ASSEMBLE project) respectively, and were provided to us as fin clips. Samples from Spain and Portugal were collected by hand-netting in tide-pools. These individuals were kept out of water for a very brief period (less than 30s), a small portion of fin dorsal fin was clipped and they were released immediately to their pool of origin. This practice has been used by our team for many years in thousands of fish and no casualties were detected, either by physical manipulation or by infections. The fish suffer less harm than if they are exposed to treatments with anesthetic which cause greater physiological disturbance. Both in Portugal and Spain there is no protection status for this species [35], [36]. At Sintra/Cascais, because the fish is locally uncommon and not enough individuals could be collected by hand net, five animals were spear-fished by an amateur diver. In Portugal there are no fishing restrictions and the diver had the necessary license to do spearfishing (246/2000). Collection of specimens complied with the current laws of each country.

### DNA Extraction, Amplification and Sequencing

Total genomic DNA was extracted from fin samples preserved in 96% ethanol with the REDExtract-N-Amp kit (Sigma-Aldrich) following the manufacturers instructions. Voucher specimens are deposited in ISPA (ethanol preserved tissues). PCR amplification of mitochondrial control region (CR) and the first intron of the nuclear S7 ribosomal protein gene (S7), were performed with the following pairs of primers: dloop – LPro1 (5′- ACTCT CACCC CTAGC TCCCA AAG - 3′) and HDL1 (5′- CCTGA AGTAG GAACC AGATG CCAG - 3′) [37] and the first intron of the S7 ribosomal protein gene – S7RPEX1F (5′- TGG CCT CTT CCT TGG CCG TC - 3′) and S7RPEX2R (5′- AAC TCG TCT GGC TTT TCG CC - 3′) [38]. PCR amplification reactions were performed in a 20 µl total-reaction volume with 10 µl of REDExtract-N-ampl PCR reaction mix (Sigma–Aldrich), 0.8 µl of each primer (10 µM), 4.4 µl of Sigma-water and 4 µl of template DNA. An initial denaturation at 94°C for 7 min was followed by 35/30 cycles (denaturation at 94°C for 30/45s, annealing at 55°C for 30/45 s, and extension at 72°C for 1 minute) and a final extension at 72°C for 7 minutes on a BioRad Mycycler thermal cycler (values CR/S7, respectively). The same primers were used for the sequencing reaction, and the PCR products were purified and sequenced in STABVIDA (http://www.stabvida.net/).

### DNA Analysis

All sequences were aligned using Clustal X [39].

ARLEQUIN software package V.3.5 [40] was used to estimate the genetic diversity within each sample, to access potential population differentiation and to perform neutrality tests. It was also used to perform analysis of molecular variance (AMOVA [41]) and to compute pairwise *F*_ST_s. Significance levels of all multiple statistical tests were corrected using the false discovery rate (FDR) approach [42] implemented in QVALUE package of software R [43]. In the case of the S7 intron the analyses were also run in ARLEQUIN, after allowing the program to reconstruct the haplotypes present, using the ELB algorithm [44].

For the CR, the spatial analysis of molecular variance (SAMOVA 1.0) [45] was used to identify groups of sampling locations which are geographically and genetically homogeneous and maximally differentiated from each other. This approach relies on a technique of analysis of molecular variance (AMOVA) [41]. However in contrast to conventional AMOVA, SAMOVA does not require that the groups’ constitution is defined *a priori,* allowing instead the groups to emerge from the data. The most likely number of groups was identify by running SAMOVA with two to six groups and choosing the partition scheme with the highest *F*_CT_ value. Because SAMOVA identified two groups which maximized *F*_CT_ (see the results section) the data of the populations included in each group were pooled and mismatch analysis [46], [47] and the Fu’s *Fs* [48] and Tajima’s D [49] tests were performed to test for possible bottlenecks and population expansion in each group. In order to compare haplotype diversities, [31] method was used for both overall diversity and pairwise comparisons between locations. To estimate nucleotide pairwise genetic distances between locations we used [50] distance, as implemented in ARLEQUIN, to correct for inherited ancestral polymorphism. An haplotype network was constructed for the CR data using Network v4.5 [51] running the maximum parsimony (MP) calculation option to eliminate superfluous nodes and links [52].

Past population demography of *T. bubalis* was inferred with the CR data using both mismatch distributions and the extended Bayesian skyline plot (BSP) [53] model as implemented in BEAST v.1.6 [54] employing the Bayesian MCMC coalescent method, a HKY+I+G model of substitution, and a strict clock. The Bayesian distribution was generated using results from five independent runs of 150 million MCMC steps obtaining effective samples sizes (ESS) of parameter estimates of over 200, with a burn-in of 10%. The time for most recent common ancestor (*t*_MRCA_) and the median and corresponding credibility intervals of the BSP were depicted using Tracer v.1.4 [55]. MtDNA control region mutation rates in fish are widely variable (e.g. 2.2–4.5%/MY between lineages for East African cichlids, Sato *et al.* 2003; 15%–20%/MY for Indo-Pacific sardines, [56]). In the absence of a clock calibration for the CR of *T. bubalis* we address the uncertainty by tentatively assuming a within lineage mutation rate of 5%/MY, which is within the range of values found by [57] after a review of CR molecular clock calibrations for several tropical Atlantic fish species. These estimations of divergence times must be interpreted with great caution. Apart from uncertainties in the molecular clock calibrations, they often assume a model of mutation-drift equilibrium which does not hold if severe bottlenecks occurred. If two sister lineages differ markedly in their population size, the one with smaller size will change at a faster rate due to increased drift. In this situation, divergence times between the two populations will be overestimated. Migration rates among populations were estimated by the MCMC approach implemented in Lamarc V. 2.1.3 [58], using 10 runs of 12 short chains of 1,000 steps and five long chains of 50,000 steps, with a burn-in of 10,000.
